# Reducing the Dispersion of Periodic Structures with Twist and Polar Glide Symmetries

**DOI:** 10.1038/s41598-017-10566-w

**Published:** 2017-08-31

**Authors:** O. Dahlberg, R. C. Mitchell-Thomas, O. Quevedo-Teruel

**Affiliations:** 1 Department of Electromagnetic Engineering, Royal Institute of Technology, SE 100 44 Stockholm, Sweden; 2 Department of Physics and Astronomy, University of Exeter, Exeter, UK

## Abstract

In this article, a number of guiding structures are proposed which take advantage of higher symmetries to vastly reduce the dispersion. These higher symmetries are obtained by executing additional geometrical operations to introduce more than one period into the unit cell of a periodic structure. The specific symmetry operations employed here are a combination of p-fold twist and polar glide. Our dispersion analysis shows that a mode in a structure possessing higher symmetries is less dispersive than in a conventional structure. It is also demonstrated that, similar to the previously studied Cartesian glide-symmetric structures, polar glide-symmetric structures also exhibit a frequency independent response. Promising applications of these structures are leaky-wave antennas which utilize the low frequency dependence.

## Introduction

A periodic structure possesses a higher symmetry when the unit cell coincides with itself after more than one linear or angular translation or reflection^1–4^. This means that a sub-unit cell, when repeated non-trivially, either with linear and/or angular translation or a reflection operation, will compose the full unit cell. In the 1960’s and 70’s, several studies were made concerning two types of higher symmetries, namely glide symmetry and twist (screw) symmetry^1–4^. In glide symmetry, the sub-unit cell is translated half its period and mirrored in a glide plane. Similarly, in a p-fold twist-symmetry, the sub-unit cell is translated and rotated around an axis. These studies uncovered the profound impact that higher symmetries have on the propagation properties of periodic structures: to reduce or even eliminate the dispersion of the lowest mode^5^, and to remove band gaps at the Brillouin zone boundary^6, 7^. A proof for the absence of band gaps in structures possessing glide symmetries is given in ref. 8, and a similar procedure can be followed for the twist-symmetric case. Similar effects have also been discussed in topological structures^9, 10^.

These induced effects have been employed in the design of a number of electromagnetic devices. Cartesian glide symmetry for 1D periodicity has been used for leaky-wave antennas in order to support both forward and backward beam radiation^11–13^. More recently, glide-symmetric metasurfaces, where translation is made in two directions, have been investigated. It was found that they can be used to reduce leakage from integrated waveguides by utilizing the symmetry induced stop band^14, 15^. Additionally, non dispersive lenses have been proposed using the wide operational bandwidth of 2D glide symmetric structures^5, 16, 17^.

In contrast to previous works focusing on Cartesian glide symmetry, in this article we investigate both twist symmetries and polar glide symmetries, as well as their combination. It has been stressed that the main benefit of employing higher symmetries in periodic structures is to vastly reduce the dispersive nature of the lowest order mode. However, it also provides the ability to increase the effective refractive index of the wave guiding structure. These characteristics of higher symmetries can find great applications in leaky-wave antennas, which can substantially benefit from the reduced dispersion and accurate control of the refractive index for less dispersive, more controllable radiation^18, 19^. Additionally, by employing higher symmetries, these effects can be achieved in fully metallic structures, reducing the losses in the system.

**Figure 1:**
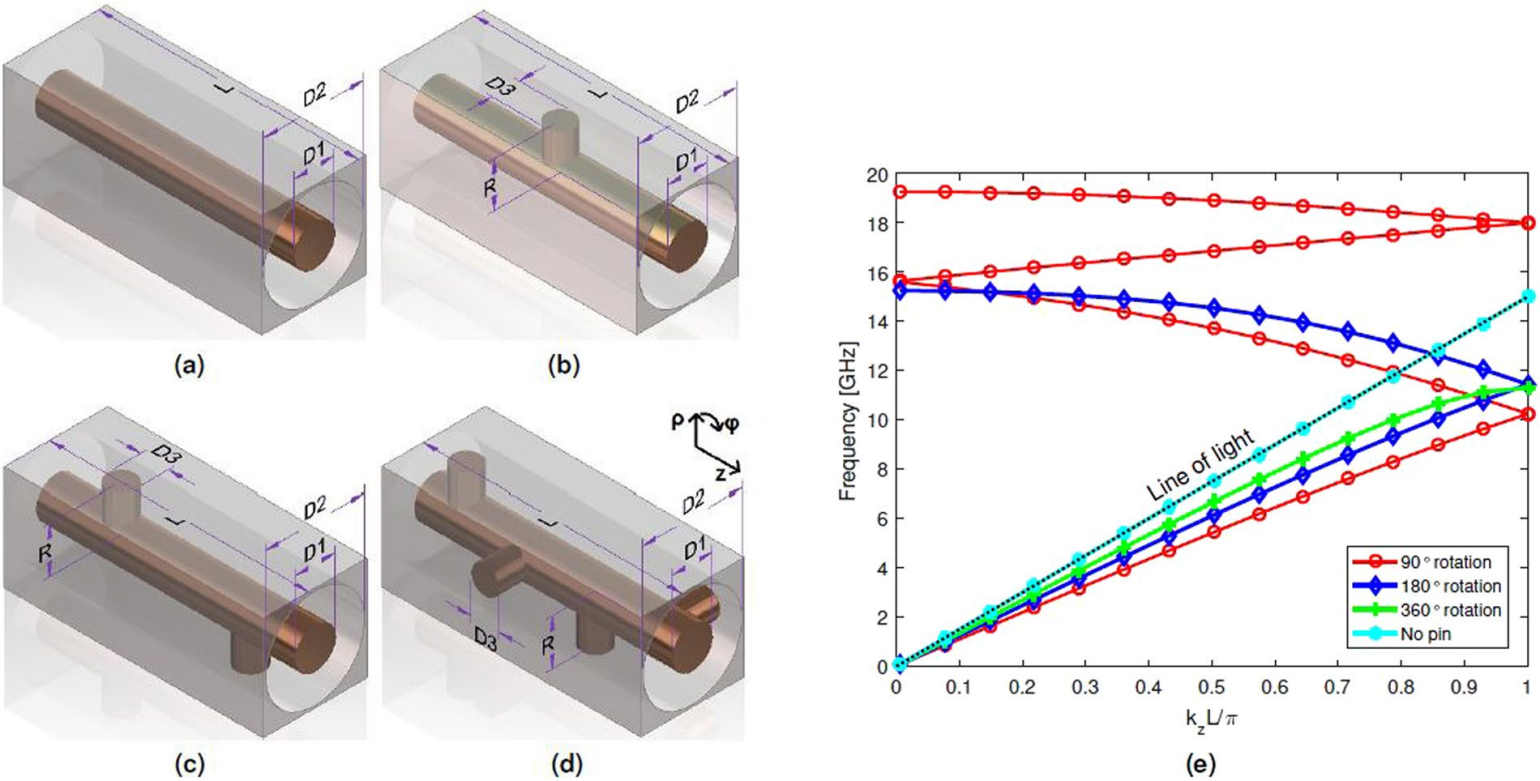
Twist-symmetric coaxial transmission lines: (**a**) Without pins, (**b**) Single pin, (**c**) 2-fold twist symmetry, (**d**) 4-fold twist symmetry and (**e**) dispersion diagram for the proposed twist-symmetric coaxial transmission lines. The selected dimensions are: D1 = 2 mm, D2 = 4.64 mm, D3 = 1.4 mm, R = 2.3 mm and L = 10 mm.

The purely twist-symmetric structures considered in this paper consist of an infinite repetition of unit cells in one direction. In a *p*-fold twist-symmetric structure, the sub-unit cell is translated a distance *L*/*p* and rotated 2*π*/*p*, where *L* is the period of the full unit cell^1^. Four different configurations are considered, all with the same period, *L*. The unit cells for all cases are depicted in Fig. 1a–d. The repetition is made along the symmetry axis of the coaxial transmission line, $\hat{z}$. The first configuration is a standard coaxial cable, composed of an inner and outer conductor, separated by air, depicted in Fig. 1a. This configuration is used as a reference for comparison. In Fig. 1b, one pin is added to the coaxial, protruding in the radial direction from the inner conductor, with an air gap between its termination and the outer conductor. This structure does not possess higher symmetries as it can be seen as merely a translation of the pin, in the *z*-direction. In Fig. 1c, a second pin is added, positioned at *φ* = 180° with respect to the first and translated by half the pitch. This constitutes a 2-fold twist-symmetric structure. Finally, in Fig. 1d, 4 pins are included where each has a rotation of 90° and a translation of a quarter of the pitch with respect to the previous pin, providing 4-fold twist symmetry.

The simulated dispersion diagram for these twist-symmetric configurations is presented in Fig. 1e. The selected dimensions are *D1* = 2 mm, *D2* = 4.64 mm, *D3* = 1.4 mm, *R* = 2.3 mm and *L* = 10 mm. This implies that the air gap between the pins and the outer conductor is 0.2 mm. The simulations are performed using the Eigenmode solver in *CST Microwave Studio*, with periodic boundary conditions in the propagation direction (*z*-direction) and PEC-boundary conditions in the transversal directions.

It has been previously discussed that structures possessing higher symmetries exhibit apparent periods that are smaller than the actual period^1^. When only a single pin is present, the mode is dispersive as it does not possess a higher symmetry (green curve). However, for the 2-fold and 4-fold twist symmetries considered here, these apparent periods are *L*/2 and *L*/4, respectively. This can be seen in Fig. 1e, where modes originating from neighbouring Brillouin zones are present. The consequence is to reduce the dispersion of the first mode due to an absence of band gap at the first Brillouin zone boundary^20, 21^. This phenomenon is similar to previously reported results from 1D- and 2D Cartesian glide-symmetric configurations^5, 11, 20^. Additionally, the effective refractive index increases with the number of pins in the same period, *L*. The same increase of refractive index could be obtained by inserting dielectrics into the coaxial but for certain applications, such as space or high frequency applications, dielectrics might not be feasible. In those cases, an all-metallic structure is advantageous.

**Figure 2:**
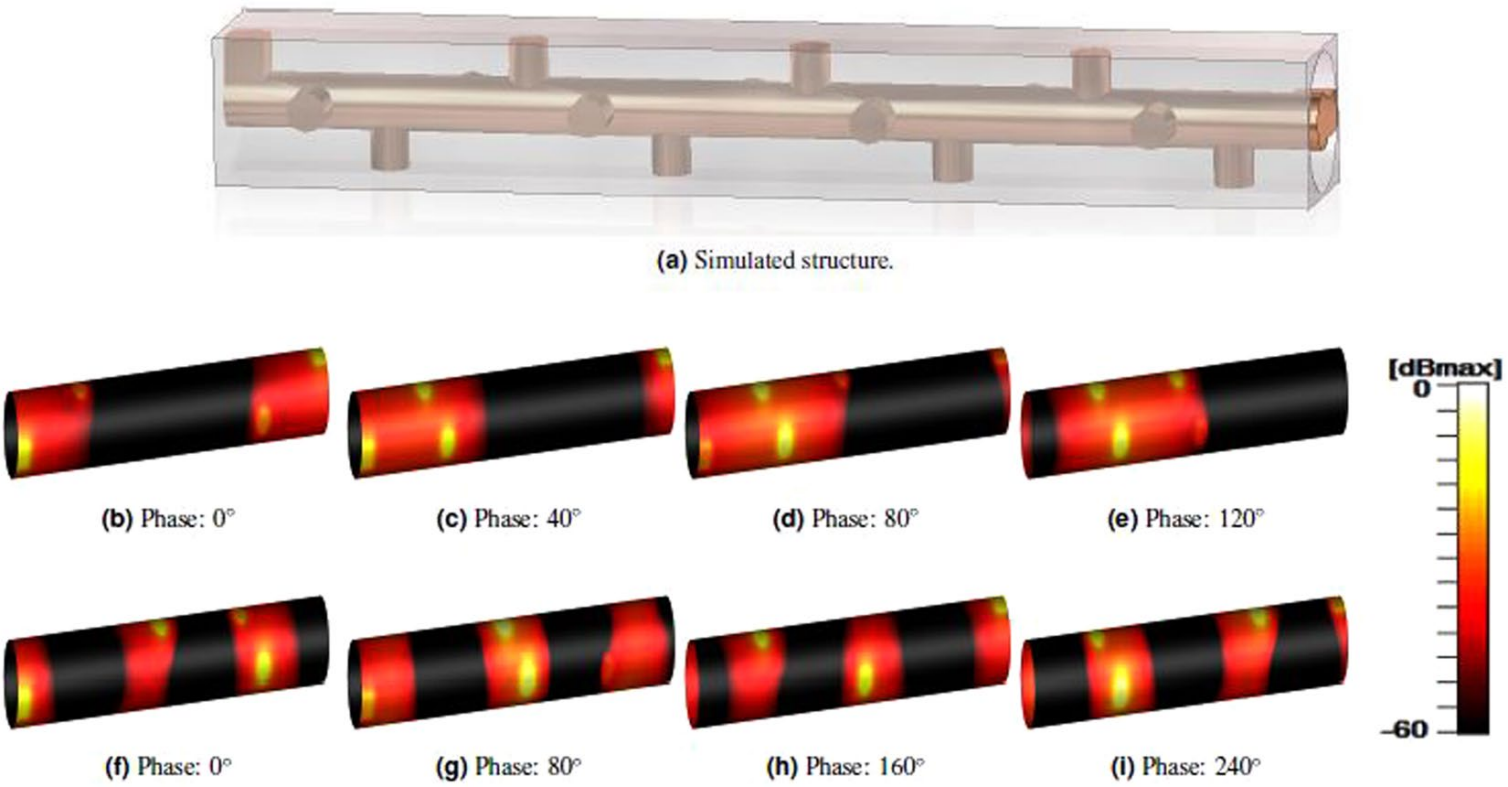
Absolute value of the electric field (normalized to the maximum value) in the 4-fold twist structure for two different frequencies, (**a**) the simulated structure with 4 unit cells, (**b**–**e**) 5.7 GHz, (**f**–**i**) 12.7 GHz. The phase is incremented with, (**b**–**e**): 40° and (**f**–**i**): 80°, for each image from left to right. The waves propagate from left to right.

A time evolution of the electric field in the 4-fold twist symmetric structure for four different times and two different frequencies (5.7 and 12.7 GHz) are illustrated in Fig. 2. The phase is incremented with 40° between each image in Fig. 2b–e, and 80° in Fig. 2f–i, due to the difference in group velocity. The majority of the electric field is concentrated between the pins and the outer conductor. This simulation was performed using the time domain solver including four unit cells. The frequencies are chosen so that the first (5.7 GHz) corresponds to the first turn in the dispersion diagram and represents the lowest order mode, and the other one (12.7 GHz) corresponds to the second turn and represents the second mode. The propagation is from left to right in all field plots. Additionally, in both modes, the energy propagates from left to right, which is the same direction as the phase. This demonstrates that the second mode is not a backward mode but a forward mode, in contrast to what was previously reported^6^.

**Figure 3:**
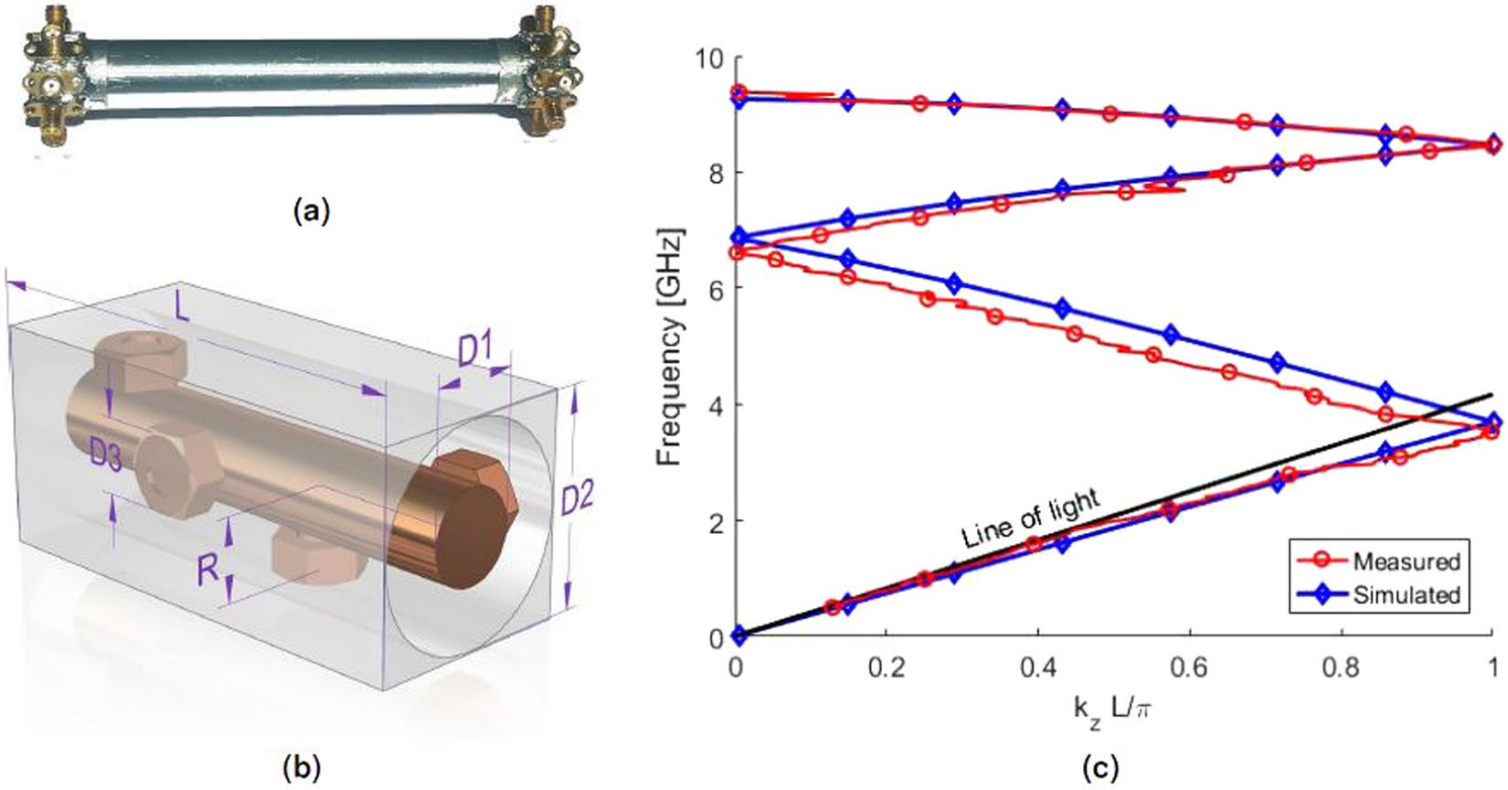
Realization of 4-fold twist symmetry, (**a**) manufactured prototype, (**b**) simulated unit cell and (**c**) simulation and measurement results.

A prototype of a 4-fold twist-symmetric structure, working up to 9 GHz is constructed and measured for verification of the simulation results. The structure is made 4 unit cells long and is fully metallic with no dielectrics. Bolts, fully screwed into the inner conductor of the structure, constitutes the pins to ensure high accuracy in the manufacturing. A picture of the prototype (4 unit cells long), together with the unit cell is presented in Fig. 3a,b. Simulated results, along with the measurements, are presented in Fig. 3c. The chosen dimensions are: *D1* = 6 mm, *D2* = 14 mm, *D3* = 5.5 mm, *R* = 5.4 mm and *L* = 36 mm. The inner conductor is suspended in air by the 8 ports at each side that are used to generate the proper TEM-mode. To obtain the dispersion diagram, the 2-port reflection and transmission coefficients are measured between all ports in transmission and one port in reception. This leads to a 9 × 9 matrix of measured S-parameters that is post-processed in Matlab, removing the effects of the ports.

**Figure 4:**
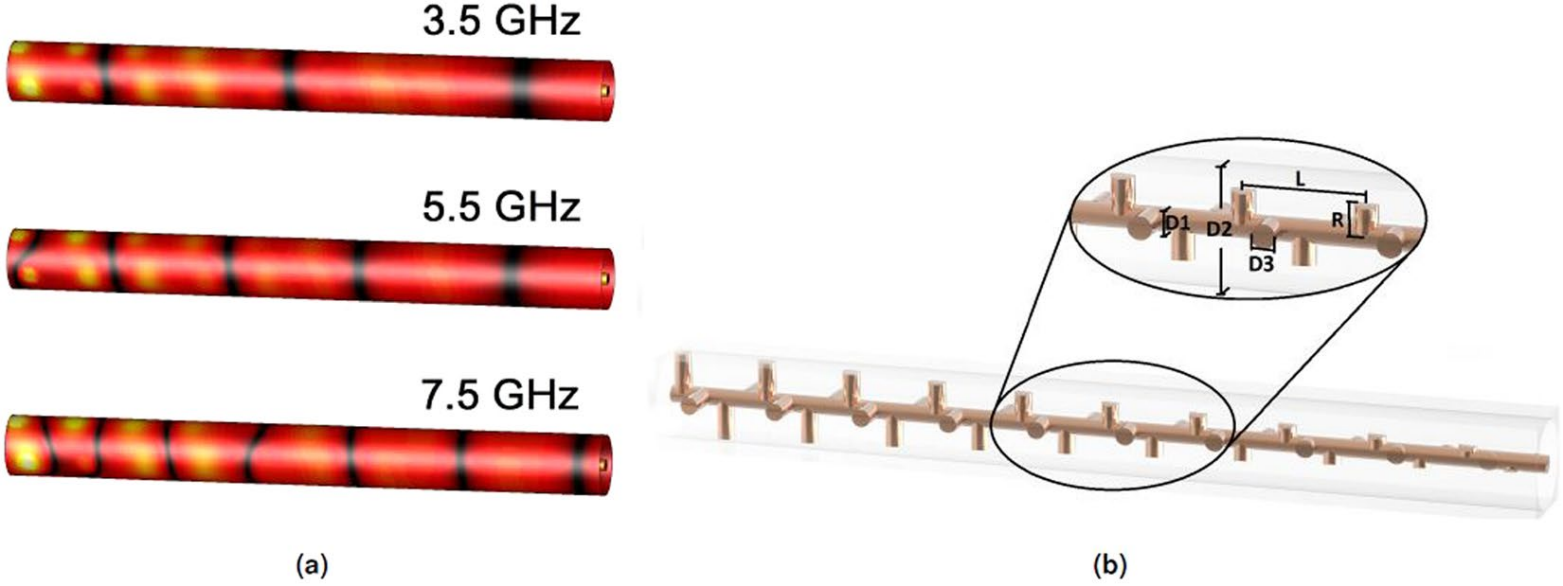
Effect of tapering the refractive index, (**a**) electric field distribution for three different frequencies and (**b**) 4-fold twist-symmetric structure with tapered pin lengths.

In applications where a grading of the refractive index along the propagation direction is desired, the dimensions of the protruding pins can be gradually changed. Contrary to using multiple dielectrics to achieve this effect, the refractive index can be tapered smoothly, resulting in very few reflections of the wave for a wide range of frequencies. Additionally, the cost of the device could potentially be lower since there is no need to manufacture multiple dielectrics. The electric field distribution, for three frequencies (3.5, 5.5 and 7.5 GHz), of a structure containing 40 pins, in a 4-fold twist configuration with pins of linearly decreasing lengths, is illustrated in Fig. 4a, with the structure in Fig. 4b. The dimensions are chosen as: *D1* = 2 mm, *D2* = 8 mm, *D3* = 1.4 mm, *R* = 4.8–0 mm and *L* = 10 mm. These dimensions results in an air gap ranging from 0.2 to 4 mm as the pin length decreases. The change in refractive index can be clearly seen as the wave length is physically shorter to the left in the figure, where the pins are longer. The change in refractive index is approximately 2:1 between the two ends of the structure, however a bigger difference could be obtained if bigger or more closely spaced pins were used.

The impact of introducing glide symmetry into a twist-symmetric structure is now introduced. Six different configurations are compared.  To obtain a twisted polar glide symmetry, three consecutive operations are carried out. The sub-unit cell is first translated linearly, followed by a rotational translation around the symmetry axis. Lastly, the translated part is mirrored in a cylindrical surface. The combination of twist- and a polar glide symmetry is realized by placing pins on both the inner and outer conductor of the coaxial. The sub-unit cell of this structure consists of two pins, one protruding from the inner conductor, and one from the outer. The pin on the outer conductor is translated half a sub-unit cell length in the *z*-direction and rotated 90° around the axis. This sub-unit cell is then repeated with 180° rotation with respect to the first to compose the full unit cell.

**Figure 5:**
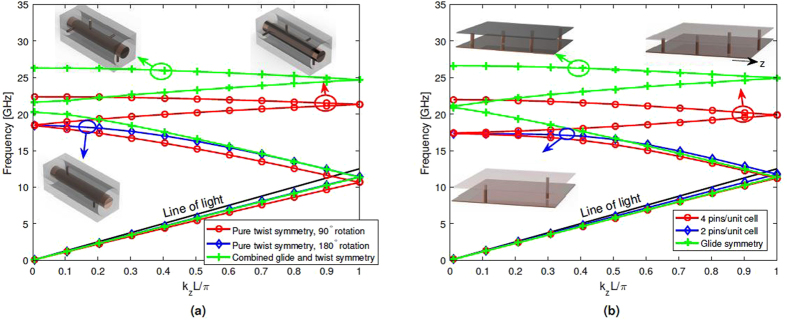
Dispersion diagram for; (**a**) the combined polar glide- and twist-symmetric case and the 2-, and 4-fold twist-symmetric cases and (**b**) the flat approximation of the the three cases.

The dispersion diagrams for this configuration are illustrated in Fig. 5a. This structure is compared with two purely twist-symmetric structures, namely the previously discussed 2- and 4-fold twists. The 2-fold twist is the same as the combined before applying polar glide. The 4-fold twist has the same number of pins as the combined. In addition to the plasmonic modes, in this case, a cavity mode exists between the pins, which has been removed for clarity.

The dimensions were chosen so that the period of the full unit cell is the same in all cases, *L* = 12 mm. The pins are of length 1.3 mm and have a radius of 0.2 mm. The air gap between pins and either the outer conductor (for the two pure twist cases) or the inner conductor (for the combined case) is 0.05 mm (red and blue curves) and 0.1 mm (green curve), respectively.

As can be seen in Fig. 5a, a narrow band gap around 20 GHz between the second and third mode is present for the combined polar glide- and twist-symmetric case. This band gap does not exist in the purely twist-symmetric cases. This is due to the fact that the influence caused by the pins connected to the inner and outer conductors is not equivalent, since the inner and outer conductors have different radii. This implies that the polar glide symmetry operation is not exact.

The comparison between the combined case and the 4-fold twist yields that the purely twist-symmetric structure slows the wave more than when a combination of symmetries are exploited. Additionally, the comparison between the 2-fold twist and the combined case shows that the propagating wave experiences a similar effect for the two structures for a wide frequency range. These studies confirm that, by introducing higher symmetries in the structure, a less dispersive mode is obtained, which is also the case when two different symmetries are used.

Finally, as the manufacturing of the proposed twist-symmetric structures is quite difficult, an approximately equivalent flat system is simulated, which uses both Cartesian glide symmetry and twist symmetry. Figure 5b shows the dispersion diagram in this equivalent flat configuration. Here, the structures have the same periodicity in the propagation direction as the coaxials in Fig. 5a. The periodicity in the direction perpendicular to propagation is 2*πr* where *r* is either the distance from the center of the coaxial to the end of a pin on the inner conductor (blue and red curve) or the average radius of the two conductors (green curve). In the flat case, the addition of Cartesian glide symmetry doubles the number of modes, without a band gap in contrast to polar glide symmetry. Except for the gap in the combined polar case, all flat structures experience very similar dispersion properties compared with the corresponding twist.

In this article, we have introduced a family of new structures for guiding waves that utilize higher symmetries, namely twist and glide symmetries. The structures are coaxial transmission lines with pins protruding in the radial direction. Notably, a reduced frequency dependence of the guiding structures is achieved. In addition to creating less dispersive modes, the band gap at the Brillouin zone boundary is absent. These structures provide an opportunity for the development of new kinds of leaky-wave antennas based on an accurate control of the non-dispersive refractive index. It has been shown that the wave speed in the structure can be tailored by changing the geometrical parameters of the system. By employing higher symmetries, we have shown that it is possible to design structures with modes that exhibit no band gaps across large frequency bands.

### Data availability statement

The data generated and analysed during the study are available from the corresponding author on reasonable request.
